# Supplementary material for: A Special Amino-Acid Formula Tailored to Boosting Cell Respiration Prevents Mitochondrial Dysfunction and Oxidative Stress Caused by Doxorubicin in Mouse Cardiomyocytes
Source: Nutrients. 2020 Jan 21;12(2):282. doi: 10.3390/nu12020282 (PMC7071384; doi:10.3390/nu12020282)
Supplement: Supplementary file 1 [file nutrients-12-00282-s001.pdf]

Supplementary Materials

A special amino-acid formula tailored to boosting cell respiration prevents mitochondrial dysfunction and oxidative stress caused by doxorubicin in mouse cardiomyocytes

Laura Tedesco[1], Fabio Rossi[1], Maurizio Ragni[1], Chiara Ruocco[1], Dario Brunetti[1], Michele O. Carruba[1], Yvan Torrente[2], Alessandra Valerio[3,*] and Enzo Nisoli[1,*]

[1]Center for Study and Research on Obesity, Department of Medical Biotechnology and Translational Medicine, University of Milan, Milan, Italy

[2]Department of Pathophysiology and Transplantation, University of Milan, Fondazione IRCCS Ca' Granda Ospedale Maggiore Policlinico, Centro Dino Ferrari, 20122, Milan, Italy

[3]Department of Molecular and Translational Medicine, University of Brescia, Brescia, Italy

* Correspondence: alessandra.valerio@unibs.it; Tel: +30 030 3717504; fax +39 030 3717529 (A.V.); enzo.nisoli@unimi.it; Tel: +39 02 50316956; fax: +39 02 50317118 (E.N.)

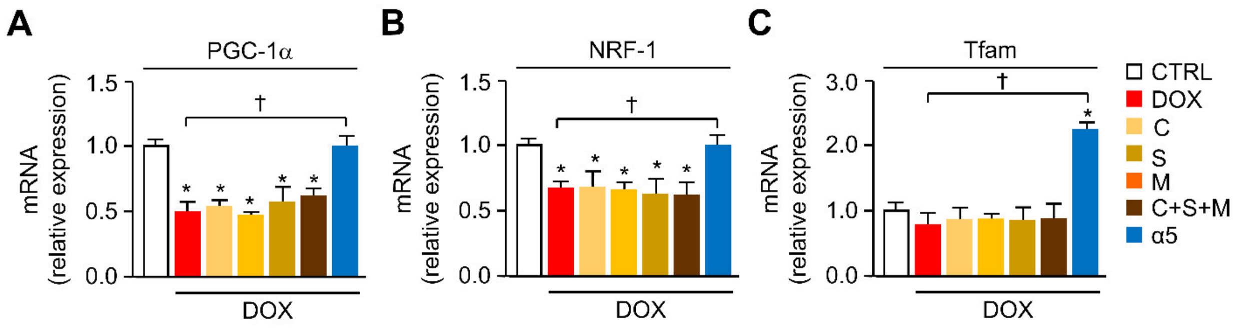

Figure S1. TCA intermediates do not prevent DOX-induced reduction of mitochondrial biogenesis genes in HL-1 cardiomyocytes. (A-C) Peroxisome proliferator-activated receptor-γ coactivator 1□ (PGC-1α) (A), nuclear respiratory factor-1 (NRF1) (B), and transcription factor A (Tfam) (C) mRNA levels were analysed by quantitative RT-PCR. Relative expression values for the untreated (CTRL) cells were taken as 1.0 (n = 3 experiments). C, citric acid; S, succinic acid, M, malic acid. *$p < 0.05$ vs. untreated cells. All data are presented as the mean ± SD.

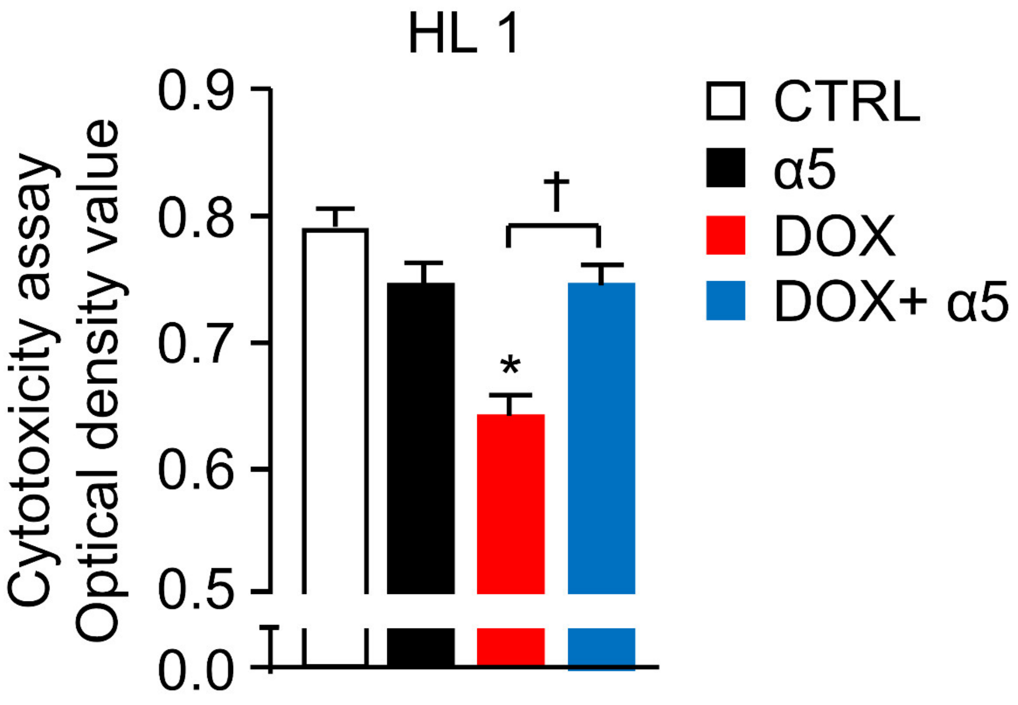

Figure S2. α5 formula prevents DOX-induced death of HL-1 cells. Cytotoxicity of HL-1 cardiomyocytes was evaluated with the MTT assay. Cells were treated with 1 % α5 for 48 h and 1 μM DOX for 16 h. *$p < 0.05$ *vs*. untreated cells; †$p < 0.05$ *vs*. DOX-treated cells. All data are presented as the mean ± SD.

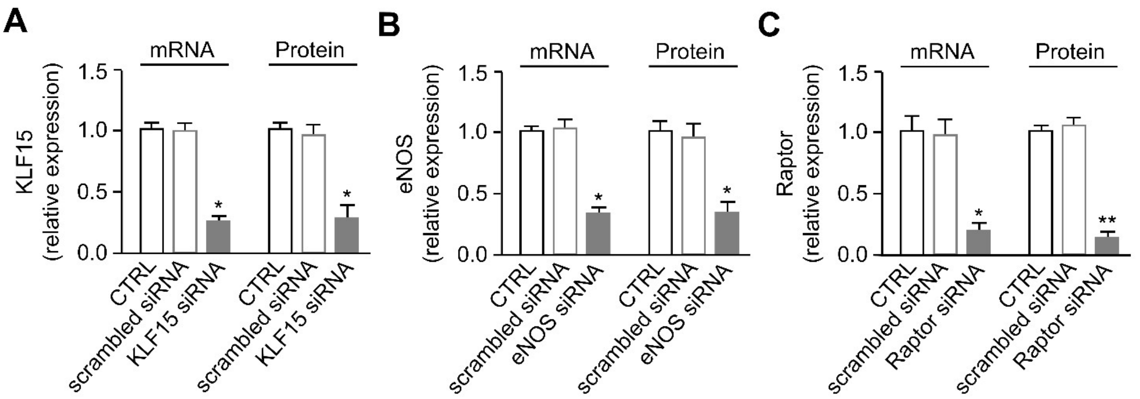

Figure S3. Specific *Klf15*, *eNOS*, and *Raptor* silencing in HL-1 cardiomyocytes. (**A-C**) *Klf15*, *eNOS*, and *Raptor* mRNA levels were analyzed by quantitative RT-PCR and KLF15, eNOS, and Raptor protein levels were detected by immunoblot analysis. Relative expression values for the untreated (CTRL) cells were taken as 1.0 (n = 5 experiments). *$p < 0.05$ *vs*. untreated cells. All data are presented as the mean ± SD.

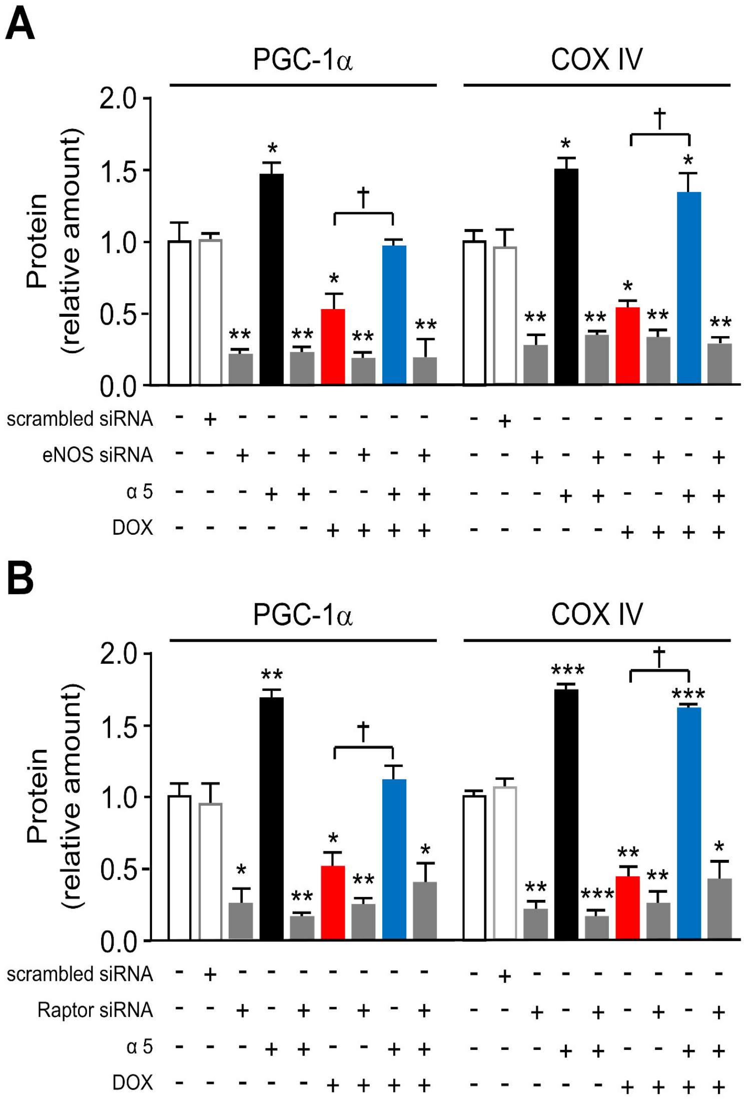

Figure S4. Specific *eNOS* and *Raptor* silencing in HL-1 cardiomyocytes. (**A**) *eNOS* and (**B**) *Raptor* mRNA levels were analyzed by quantitative RT-PCR, and eNOS and Raptor protein levels were detected by immunoblot analysis. Relative expression values for the untreated (CTRL) cells were taken as 1.0 ($n = 3$ experiments). $^*p < 0.05$, $^{**}p < 0.01$, and $^{***}p < 0.001$ *vs*. untreated cells; $^{\dagger}p < 0.05$ *vs*. DOX-treated cells. All data are presented as the mean ± SD.

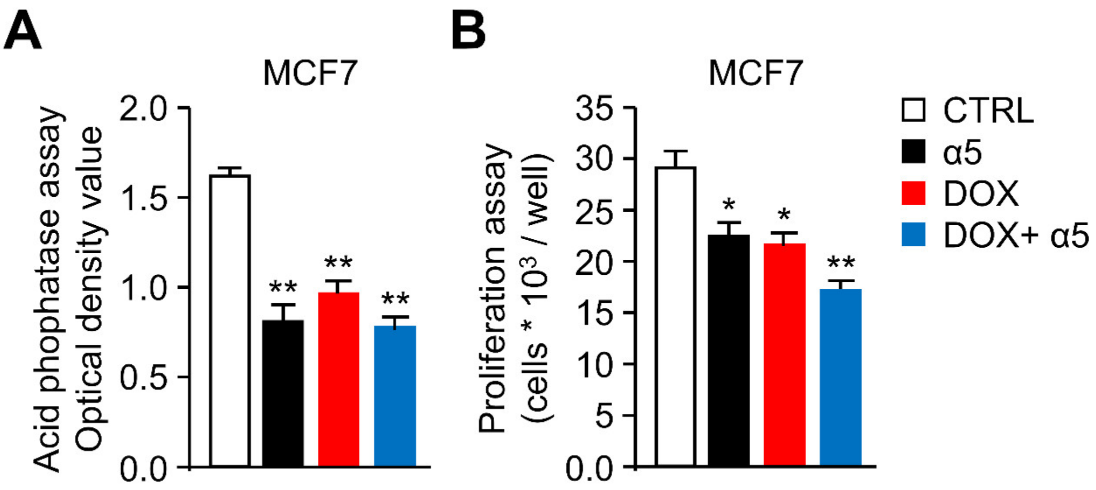

Figure S5. MCF7 breast cancer cell proliferation. The anti-proliferative effect of DOX remained unchanged in the MCF7 cells in the presence of the amino acid mixture. (**A**) Acid phosphatase assay: cells (5,000-20,000/well in 96-well plates) were treated with 1 % α5 for 48 h and 1 μM DOX for 16 h. (**B**) Proliferation assay: cells (50,000/well in 12-well plates) were treated as in (A) and Trypan blue exclusion assay was used. $n = 3$ experiments. $^{*}p < 0.05$ and $^{**}p < 0.01$ *vs.* untreated cells. All data are presented as the mean ± SD
